# Comparative Study of Red and Grey Selenium Nanoparticles on Organ-Specific Selenium Deposition and Growth Performance in Japanese Quails

**DOI:** 10.3390/nano15110801

**Published:** 2025-05-27

**Authors:** Aya Ferroudj, Arjun Muthu, Daniella Sári, Gréta Törős, Áron Beni, Levente Czeglédi, Renáta Knop, Hassan El-Ramady, József Prokisch

**Affiliations:** 1 Nanofood Laboratory, Department of Animal Husbandry, Institute of Animal Science, Biotechnology and Nature Conservation, Faculty of Agricultural and Food Sciences and Environmental Management, University of Debrecen, 138 Böszörményi Street, 4032 Debrecen, Hungary; ferroudj.aya@agr.unideb.hu (A.F.); arjunvmuthu@gmail.com (A.M.); saridaniella91@gmail.com (D.S.); toros.greta@agr.unideb.hu (G.T.); 2 Doctoral School of Animal Husbandry, Faculty of Agriculture and Food Sciences and Environmental Management, University of Debrecen, 4032 Debrecen, Hungary; czegledi@agr.unideb.hu (L.C.); dr.knop.renata@agr.unideb.hu (R.K.); 3 Institute of Agricultural Chemistry and Soil Science, Faculty of Agricultural and Food Sciences and Environmental Management, University of Debrecen, 138 Böszörményi Street, 4032 Debrecen, Hungary; beniaron@agr.unideb.hu; 4 Soil and Water Department, Faculty of Agriculture, Kafrelsheikh University, Kafr El-Sheikh 33516, Egypt; hassan.elramady@agr.kfs.edu.eg

**Keywords:** selenium, nanoparticles, amorphous, crystallin, avian, bioavailability, tissue, quail, growth

## Abstract

Selenium (Se) is an essential trace element required for various physiological functions in agriculture. Nanotechnology is applied to produce selenium nanoparticles (SeNPs) that offer new advantages, enhancing their bioavailability and reducing toxicity. To further improve the stability of Se nanoelements in the poultry industry, the grey form of Se was recently offered as a potential alternative. However, its impact on bioaccessibility, metabolism, and overall animal efficiency remains undetermined. This study investigates the impact of red and grey SeNPs on Se content in the liver, blood cellular fraction (BCF), kidney, testis, and eyes, as well as the feed intake (FI) and growth performance, of adult Japanese quails. Adult quails were randomly assigned to five groups: a control (C0) and four groups receiving either red or grey Se nanoparticles (SeNPs) at 0.05 or 0.5 mg/kg, in addition to the basal diet which already contained 0.042 mg/kg Se from the premix, resulting in total Se contents of approximately 0.092 and 0.542 mg/kg in the treatment groups (T1–T4), with four replicates per group. The growth performance of quails fed with nano-Se-supplemented diets showed significant variation across groups (*p* < 0.05), with body weight differing by up to 20% between the highest performing group (T2) and the lowest (T1). FI showed no significant differences across groups. The results indicated that Se accumulation differed significantly between treatments. The selenium levels in the liver increased in a dose-dependent manner, with the highest accumulation observed in T4 (0.5 mg/kg grey SeNPs), at 42% above control levels. This pattern suggests that the liver is a primary organ for selenium storage and metabolism. The greatest Se content in BCFs was recorded in the groups that received grey selenium (T3 and T4) and red selenium at high concentrations (T2), while the group given red selenium at low concentrations (T1) and the control (C0) had the lowest Se accumulation. In the kidney tissues and testis, the Se content exhibited no significant differences between the treated groups and the control. The observed variations in the eye and breast muscle Se content among treatment groups reflect the differences in selenium bioavailability, metabolism, and tissue-specific regulatory mechanisms. These findings demonstrate that grey SeNPs can significantly elevate Se bioavailability in quails, particularly in target organs, and enhance the growth performance without notable changes in feed intake. This highlights the potential of SeNPs in enhancing quail nutrition, although further research is needed to establish optimal dosing strategies for safe, effective use.

## 1. Introduction

Selenium (Se) is an essential trace element required for various physiological functions in poultry, including growth, metabolism, immune response, and antioxidant defence [1,2]. As a key component of selenoproteins such as glutathione peroxidase (GPx), selenium plays a crucial role in protecting cells from oxidative stress and maintaining overall health [3,4,5]. Selenium deficiency can lead to biomass loss and reduced growth performance in poultry. The antioxidant system relies on adequate selenium levels for proper functioning, so the activities of selenoenzymes such as glutathione peroxidase (GPx), superoxide dismutase (SOD), and catalase (CAT) can be limited with low Se content, inducing oxidative stress and general inflammation in several vital organs and disrupting thyroid function, immunity, growth, sexual activity, and general body vitality [1,2,6,7,8]. While inorganic and organic selenium sources have been widely used in poultry, ruminants, and aquaculture species nutrition, concerns regarding their variable bioavailability and potential toxicity at high dosages have been raised [9,10,11,12]. Inorganic Se (selenite and selenate) has a lower bioaccessibility in vital liquids and high toxicity because of its high susceptibility to chemical reactions with other minerals, which convert it to inutile forms that are excreted via exhalation and urine or require a long path to reach the useful form (selenide) [13,14]. Significant health concerns can be associated with high exposure to these kinds of selenium and exceeding upper limits, inducing toxicity symptoms. They can promote severe gastrointestinal difficulties, neurological illness, and metabolic disorders, even organ failure (cardiac muscle, kidney, etc.) [15,16,17]. The chronic overconsumption of selenium, including its organic forms, is linked to selenosis, which causes patients to experience hair loss, nail loss, garlic breath, and skin irritation [18,19] and can lead to cancer and diabetes [20,21].

To address these limitations, nano-selenium (SeNPs) has emerged as a promising alternative, offering higher bioavailability, reduced toxicity, and enhanced biological reactivity [22]. Se at the nanoscale provides high absorption and enhanced bio-utilization because of its large surface area [4,23,24], which increases the interaction with the cellular gut wall, and its small size allows instant diffusion into the blood stream and tissues [25]. However, the toxicity threshold is wider compared to organic and inorganic selenium. This makes the nanoparticles (NPs) of Se supplementation more efficient [24,25,26]. Among different SeNP forms, red and grey SeNPs exhibit structural and physicochemical differences; red amorphous SeNPs quickly transition into a grey hexagonal structure (grey SeNPs) in liquid, under atmospheric conditions [27], potentially influencing their absorption and physiological effects. in particular, grey SeNPs have demonstrated greater stability [27], likely due to their thermodynamic stability under ambient conditions and the ability to maintain their crystalline structure under extreme thermal environments [23,28]. But their potential benefits in livestock feeding and metabolic efficiency remain unexplored. A comprehensive analysis of selenium distribution in vital organs—including the liver, kidneys, testes, blood, and ocular tissues—is crucial for optimizing its physiological efficacy and reducing potential toxicity risks associated with nanoparticle supplementation. Japanese quails (*Coturnix japonica*) were chosen due to their established use in nutritional and toxicological studies, characterized by rapid growth, well-documented physiology, and ease of handling in laboratory settings [5,29]. Their metabolic similarity to other poultry species and sensitivity to micronutrient variations make them suitable model organisms for studying trace element bioavailability. The objective of this study was to evaluate the comparative effects of dietary supplementation with red and grey selenium nanoparticles, at two different dosages (0.05 and 0.5 mg/kg), on growth performance, organ development, and selenium accumulation in specific tissues (liver, kidney, spleen, blood, testis, eyes, and breast muscle) in adult male Japanese quails.

## 2. Materials and Methods

This study was conducted at the University of Debrecen, Faculty of Agricultural and Food Sciences and Environmental Management, Institute of Animal Science, Biotechnology and Nature Conservation, Department of Animal Husbandry, Nanofood laboratory, Hungary. It was approved by the institutional Ethics Committee of the University of Debrecen (ethical permission number: 4/2021/DEMÁB). All methods were performed following the relevant guidelines and regulations.

### 2.1. Reagents

Sodium selenite, vitamin C, nitric acid 65% (AR grade), hydrogen peroxide, and hydrochloric acid 37% (AR grade) were obtained from VWR, International Ltd. (Lutterworth, Leics, UK). Sodium borohydride 98% (AR grade) was purchased from Acros Organics (Geel, Belgium).

### 2.2. Selenium Nanoparticle Preparation and Characterization

Red and grey selenium nanoparticles (SeNPs) were synthesized following the method of [27]. Red SeNPs were obtained by reducing sodium selenite (Na_2_SeO_3_) with 1% ascorbic acid at room temperature for 30 min, yielding a red colloidal suspension. Grey SeNPs were produced by thermally transforming red SeNPs at 85 °C for 12 h to induce phase transition into the hexagonal crystalline structure. As previously characterized by [27], the red SeNPs had an average particle size of 80–120 nm, while grey SeNPs ranged from 90 to 150 nm, as determined by transmission electron microscopy (TEM) and dynamic light scattering (DLS). UV-Vis spectrophotometry confirmed the purity of both nanoparticle types to be above 95%, with no detectable residual selenium species such as selenite or selenate. These physicochemical properties were validated in our laboratory using the same synthesis protocol.

### 2.3. Experimental Design

A total of 20 adult male Japanese quails (*Coturnix japonica*), 11 weeks of age, were used in a 28-day feeding trial. Birds were individually housed in wire cages under standardized environmental conditions (temperature: 25 ± 2 °C; photoperiod: 16 h light/8 h dark) to allow precise monitoring of feed intake and health status. All birds had free access to feed and water throughout the experimental period, with approximately 18 g of feed offered per bird per day. Quails were randomly allocated into five dietary treatment groups (*n* = 4 per group) based on initial body weight to ensure uniformity among groups. The treatments were as follows:

o C0 (Control): Basal diet without selenium nanoparticle (SeNP) supplementation.
o T1: Basal diet supplemented with 0.05 mg/kg red SeNPs.
o T2: Basal diet supplemented with 0.5 mg/kg red SeNPs.
o T3: Basal diet supplemented with 0.05 mg/kg grey SeNPs.
o T4: Basal diet supplemented with 0.5 mg/kg grey SeNPs.

**Figure 1:**
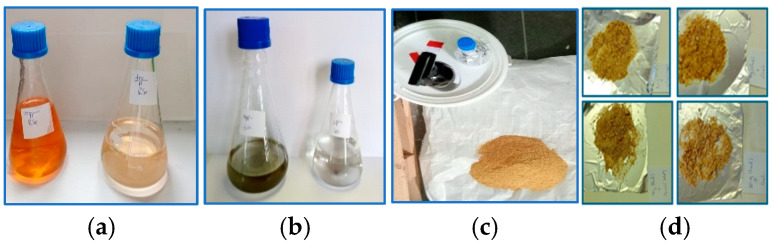
Preparation and incorporation of selenium nanoparticles in experimental diets: (**a**) synthesis of red selenium nanoparticles; (**b**) synthesis of grey selenium nanoparticles; (**c**) preparation of selenium-supplemented feed; (**d**) homogenization of selenium nanoparticles in feed samples.

The basal diet (Table 1) was formulated using soybean, corn, wheat, and sunflower oil taking into account the nutrient requirements of breeder quails according to [30]. The premix included in the basal diet provided a background selenium content of 0.042 mg/kg. Consequently, the total selenium content in the diets was estimated to be 0.042 mg/kg (C0), 0.092 mg/kg (T1 and T3), and 0.542 mg/kg (T2 and T4), as shown in Figure 1.

### 2.4. Growth Performance

Body weight (BW) and feed intake (FI) were recorded daily. Total BW and total FI were used to evaluate growth performance.

### 2.5. Tissue Sampling

Tissue samples (*n* = 4 per treatment group) were euthanized at 28 days. Liver and spleen weights were measured immediately after scarification. Tissue samples were taken from the following organs: liver, spleen, kidney, blood, testis, breast, and eyes, washed using phosphate-buffered saline solution (PBS), and were stored at −80 °C until analyzed. The samples of 0.5 g were digested with 2.25 mL of concentrated HNO_3_ (65%) and 6.75 mL of concentrated HCl (37%), then heated for 4h at 80 °C. Selenium measurements were achieved via atomic fluorescence spectrophotometer (AFS) Millennium Excalibur 10.055 (PSA, Orpington, UK).

### 2.6. Statistical Analyses

All statistical tests were conducted using the MINITAB (version 19.1, 64-bit, Minitab LLC, State College, PA, USA), and the results are reported as mean values ± standard error of the mean. The differences between the Se supplementation groups were analyzed by a one-way analysis of variance (ANOVA), followed by a Fisher pairwise comparisons test when a statistically significant (*p* < 0.05) result was observed among the different treatment groups. ChatGPT (OpenAI, GPT-4) has been used for proofreading the manuscript.

## 3. Results

### 3.1. Growth Performance

**Figure 2:**
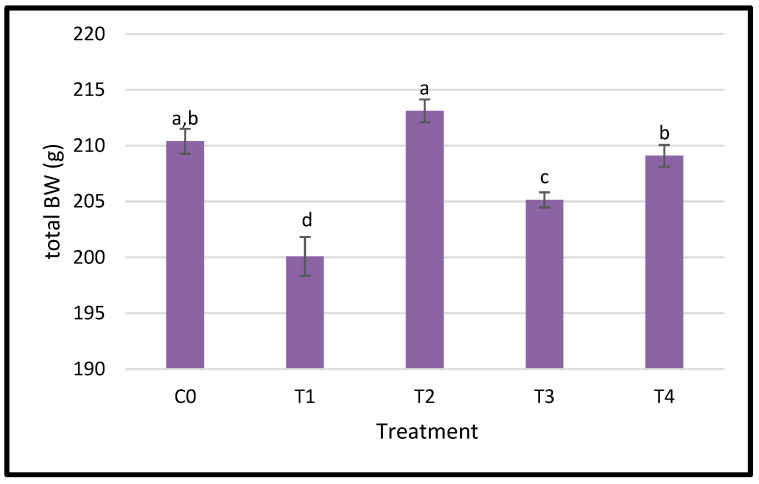
Influence of different levels of selenium nanoparticle (SeNP) dietary supplementation on the body weight (g) ± SEM of adult Japanese quails. Means with the differing letter are significantly different (*p* < 0.05). C0: control; T1: 0.05 mg/kg red SeNP; T2: 0.5 mg/kg red SeNP; T3: 0.05 mg/kg grey SeNP; T4: 0.5 mg/kg grey SeNP.

Figure 2 exhibits the growth performance of regularly fed diets supplemented with varying amounts of dietary nano-Se. The body weight was significantly affected by the nanoparticle treatments, as indicated by the statistical differences between groups; the group with treatment 2 (0.5 red nano-Se) achieved the highest BW average following by the control group and group T4 (0.5 grey nano-Se) that showed an overlapping performance between the control group and T3 group (0.05 grey nano-Se) and lowest BW average was recorded in T1 (0.05 red nano-Se).

**Figure 3:**
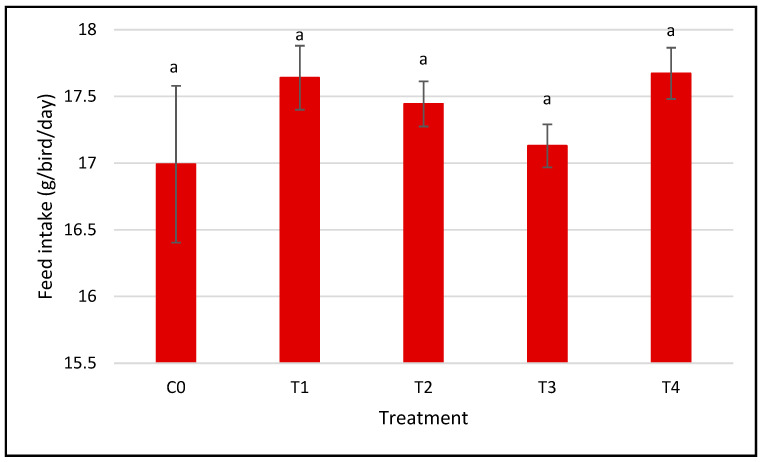
Impact of different doses of selenium nanoparticle (SeNP) dietary supplementation on the total feed intake (g) ± SEM of adult Japanese quails. Means with the same letter are not significantly different (*p* > 0.05). C0: control; T1: 0.05 mg/kg red SeNP; T2: 0.5 mg/kg red SeNP; T3: 0.05 mg/kg grey SeNP; T4: 0.5 mg/kg grey SeNP.

The feed intake was measured daily during the 28-day trial for the five groups treated with SeNP supplements. Figure 3 shows the total feed intake, which indicates that the feed intake was not significantly affected by the various selenium doses and forms. All groups consumed comparable amounts of food, which indicates that Se did not influence the birds’ appetite and feed consumption patterns. Instead, the improvements in BW are likely due to enhanced nutrient utilization and metabolic efficiency, rather than increased consumption. SeNPs are known to improve antioxidant defense, support thyroid hormone metabolism, and enhance selenoprotein synthesis—all of which are critical for optimal growth and physiological function. This is consistent with the findings of [26,31], who reported that nano-Se supplementation enhances antioxidant status, enzyme activity, and feed conversion efficiency. This divergence between feed intake and growth suggests that nano-selenium may help birds better utilize consumed nutrients, improving overall performance.

### 3.2. Organ’s Indices

**Figure 4:**
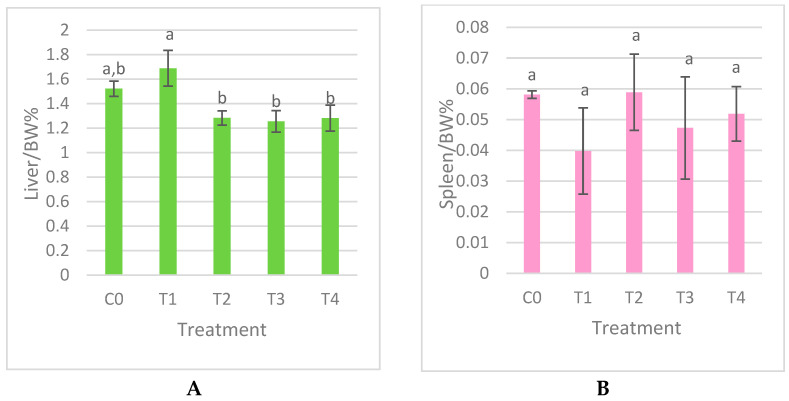
Effects of dietary selenium nanoparticle supplementation on liver (**A**) and spleen (**B**) weights (relative to body weight) in adult Japanese quails ± SEM. Means with the same superscript are not significantly different (*p* > 0.05), while means with different letters are significantly different (*p* < 0.05). C0: control; T1: 0.05 mg/kg red SeNP; T2: 0.5 mg/kg red SeNP; T3:0.05 mg/kg grey SeNP; T4: 0.5 mg/kg grey SeNP.

In our study, the liver index presented in Figure 4A demonstrates a notable variation among the groups treated with SeNPs (*p* < 0.05). The highest values were recorded in treatment 1 (0.05 red nano-Se) followed by the lowest values in control 0, treatment 2 (0.5 red nano-Se) and T3 (0.05 grey nano-Se); meanwhile, the group T4 (0.5 grey nano-Se) shared grouping with the control and treatment 2 groups. Figure 4B shows the spleen relative weights where there are no significant differences across all treatments (*p* > 0.05).

### 3.3. Selenium Deposition

**Figure 5:**
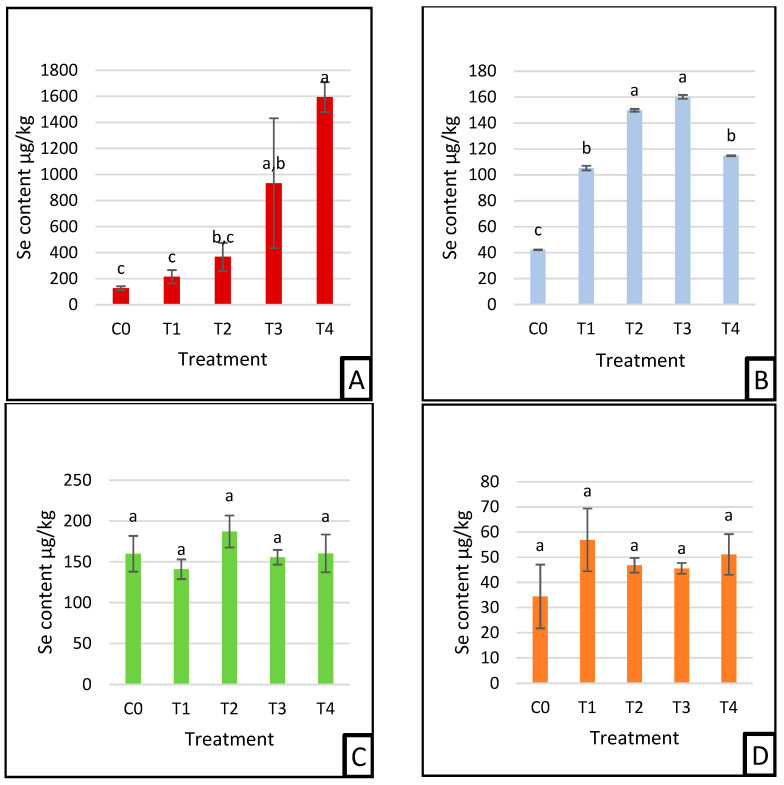

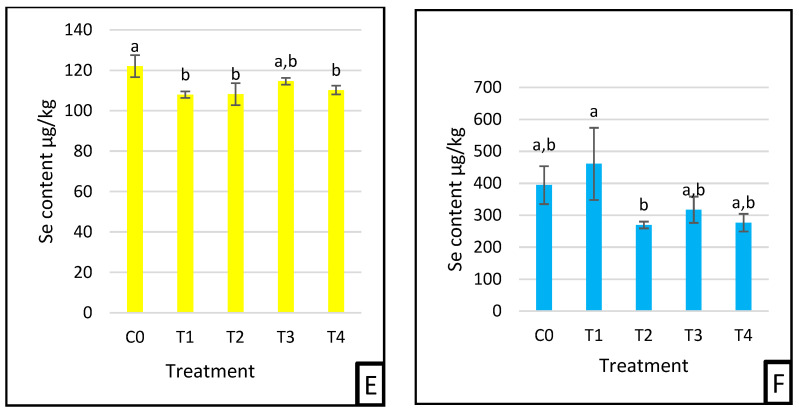
Nanoparticle of selenium distribution on liver (**A**), blood cellular fraction (**B**), kidneys (**C**), testis (**D**), eyes (**E**), breast (**F**). Means ± SEM with the same superscript are not significantly different (*p* > 0.05), while means with different letters are significantly different (*p* < 0.05). C0: control; T1: 0.05 mg/kg red SeNP; T2: 0.5 mg/kg red SeNP; T3: 0.05 mg/kg grey SeNP; T4: 0.5 mg/kg grey SeNP.

Selenium (Se) distribution varied across tissues depending on the treatment group and tissue type (Figure 5A–F).

In the liver (Figure 5A), the selenium content was highest in T4 and lowest in the control group (C0). Significant differences were observed among groups, with the Se level in T4 significantly higher than all others (*p* < 0.05). The liver Se concentrations followed the pattern: control (C0), red Se 0.05 mg/kg (T1), red Se 0.5 mg/kg (T2), grey Se 0.05 mg/kg (T3), and grey Se 0.5 mg/kg (T4), indicating a dose-dependent increase, particularly with grey SeNP supplementation. In the blood cellular fraction (Figure 5B), the highest Se concentrations were observed in T2 and T3, both significantly higher than the control (C0), T1, and T4. The control group exhibited the lowest Se level among all groups. This suggests an enhanced selenium uptake in red SeNP-treated groups at high doses and grey SeNP-treated groups at low doses. In the kidney (Figure 5C) and testis (Figure 5D), selenium content showed no statistically significant differences among the treatment groups. This indicates stable selenium deposition in these organs, regardless of SeNP form or concentration. In the eye tissue (Figure 5E), the control group had the highest selenium concentration, which was significantly different from all the Se-supplemented groups. The lowest Se levels were observed in T1, T2, and T4, while T3 showed intermediate levels, suggesting a potential decrease in ocular selenium levels following SeNP supplementation. Selenium levels in breast muscle (Figure 5F) were highest in T1, followed by the control group, while T2 showed the lowest levels (*p* < 0.05), indicating a tissue-specific sensitivity to the form and dose of SeNPs.

## 4. Discussion

Nano-selenium supplementation of Japanese quails (*Coturnix japonica*) with 0.5 mg/kg in red and grey forms and with 0.05 mg/kg grey Se was effective in increasing the growth performance without showing any improvement in feed consumption, indicating that these doses of selenium contributed progressively to body weight gain. This agrees with the research results of [4,5,32,33,34] which revealed better growth rates in avian species: quails, chickens, and broilers treated with nanoparticles of Se between the ranges of 0.2; 0.3; 0.4; and 0.6 mg/kg. Meanwhile, the recommended level of Se is 0.15 mg/kg in poultry feeding [30]. However, 0.05 mg/kg in the red selenium group showed signs of Se deficiency such as reduced body weight boost. It was found that the nanoelement Se has an identical effect in layer chicks where the growth performance was impaired at a level of 0.3 mg/kg [3]. The body weight alterations were most observed due to improved nutrient utilization rather than feed consumption. A comparable trend has been reported by [29,34] where selenium treatment enhanced feed efficiency while not affecting total feed intake across all treatments.

The liver or spleen index is the relative organ weight to the bird body weight; these are markers that reflect the morphological and functional changes in organs and are used to evaluate the toxicity of the supplements [11,35]. In a similar study, the evaluation was of the glycine nano-selenium effect in the immunity of mice where the supplement indicated no significant difference in the liver, spleen, and lung indices that demonstrate that these nanoparticles had no poisoning effect [35]. The organs’ relative weight alterations may appear because of the bird’s body weight changes. According to [12], the heart, liver, and digestive organs were not impacted by the nano-Se and inorganic selenium in broiler quails. However, the younger quails in the fattening period can be affected by dietary chemical nano-selenium and it increased the liver index [22]. The spleen index remained within the normal range [0.04–0.06]% indicating that SeNPs did not cause notable damage or affect the immune response [31]. An Se additive had no effect on the spleen relative weight of growing Japanese quails but showed a significant impact on the immune system [29]. In contrast, the Japanese quail chicks demonstrated a noticeable elevation of lymphoid organ weights [1.8–3]% due to their enhanced absorption and targeted tissue delivery [12].

The results of our study indicate that the selenium levels administered were well within the safe range for poultry. The toxicity threshold varies depending on the chemical form and species, with poultry generally exhibiting toxic effects at dietary selenium levels exceeding 0.15–0.50 mg/kg, as reported in [30,36]. Organic selenium sources, such as selenomethionine and selenium nanoparticles, are typically less toxic than inorganic forms like sodium selenite and selenate [37]. Given that the selenium concentrations in our experimental groups remained significantly below the toxic range, it is unlikely that any adverse effects observed were due to selenium toxicity. Instead, the differences in physiological responses can be attributed to variations in selenium bioavailability and metabolism. The findings in Figure 5A reveal that selenium levels in the liver increase in a dose-dependent effect, with the highest accumulation observed in the grey selenium groups (T3, T4), followed by red selenium (T2). This pattern suggests that the liver, a primary organ for selenium storage and metabolism [5,32], effectively retained selenium from both grey and red selenium nanoparticles, particularly at higher doses. The lower selenium levels in the control and red selenium (T1) groups indicate minimal selenium availability for hepatic accumulation due to insufficient supplementation. The pronounced retention in the grey-treated groups could be attributed to higher bioavailability or efficient uptake of grey selenium nanoparticles compared to red selenium, possibly due to differences in the surface chemistry and particle stability [38]. Additionally, the liver’s ability to regulate selenium levels through storage and selenoproteins synthesis and activities, such as glutathione peroxidases (GPxs) may explain the significantly higher selenium deposition in higher-dose treatments without any poisoning signs compared to other selenium species [3,4,39]. The selenium nanoparticle supplementation considerably boosted their incorporation into the blood cellular fraction, which supports the earlier research showing selenium’s function in regulating oxidative stress and erythropoiesis [5]. The highest levels were observed in the grey and red selenium-supplemented groups. This suggests that both grey and red selenium nanoparticles delivered substantially absorbed selenium to enhance uptake into the red blood cells (RBCs), likely due to their role in synthesizing selenoproteins like glutathione peroxidase (GPx) [5,9,40]. The control group exhibited the lowest selenium content, confirming the limited selenium availability in a non-supplemented diet. Selenium deposition varied significantly among the treatment groups, where red selenium at a high dose and grey selenium at a low dose exhibited similar selenium incorporation, suggesting that grey SeNPs might have a higher bioavailability, allowing lower doses to achieve comparable uptake. Conversely, low-dose red selenium and high-dose grey selenium also showed similar deposition levels, indicating a potential saturation threshold where excess dietary selenium, particularly in the more bioavailable grey SeNP form, did not further enhance tissue accumulation. These results highlight that selenium homeostasis plays a crucial role in regulating absorption and storage, preventing excessive accumulation beyond optimal physiological levels. The observed differences between selenium forms may be attributed to variations in structural stability, absorption efficiency, and metabolic pathways, where grey SeNPs exhibit enhanced bioavailability compared to red SeNPs. While this study offers valuable insights into selenium deposition patterns and relative bioavailability of red and grey SeNPs, it did not include measurements of key selenoenzyme activities such as glutathione peroxidase (GPx), superoxide dismutase (SOD), or catalase (CAT), which play critical roles in oxidative stress regulation. Due to resource constraints, these functional biomarkers could not be assessed. Nevertheless, future research should prioritize the evaluation of these enzymes to understand better the physiological impacts of SeNP uptake and their potential antioxidative benefits. However, the lack of substantial differences in selenium content in the kidneys and testis suggests a uniform distribution and systemic controls that prevent excessive deposits in these organs. The kidneys are key organs for selenium homeostasis, balancing absorption and excretion to prevent toxicity [16]. The large nanostructures can occur renal damage because of the prolonged retention in the kidneys [10]. As a preventative measure, the selenium content remains stable within the tolerable range in the kidney due to its immediate breakdown, as reported in [34]. The constant is marked in the genital organs as well, suggesting that the selenium uptake did not exceed the threshold for further storage in the testis of the quails. Furthermore, [10] reported that nano-selenium enhances Se content in bucks testis, improving the semen quality and the fertility of bulls and rams. Additionally, in previous applications of nano-selenium in Japanese quail chicks, the dietary supplement significantly increased Se abundance in their testis and ovaries [41]. This may suggest that Se distribution is tissue-specific and influenced by the physiological priorities (age and species) and the limitation in retention in these sensitive organs may indicate a controlled protective mechanism against any toxicity and poisoning. The selenium content in the eyes exhibited a regulated pattern (Figure 5E), with the control group showing the highest levels, significantly differing from most supplemented groups. Both red selenium at low and high doses, as well as grey selenium at a high dose, had similarly lower selenium levels, suggesting a saturation mechanism that limits excessive accumulation in the ocular tissues. The low-dose grey selenium group showed an intermediate position, not significantly different from either the control or supplemented groups, indicating potential variations in selenium uptake efficiency. These findings suggest that selenium homeostasis in the eye is tightly controlled, preventing excessive deposition regardless of dietary supplementation, possibly through endogenous regulatory mechanisms. In our findings, the selenium levels in quail eyes remained constant within the different forms and dosages, which is consistent with the study of [42], which reported a low and similar Se accumulation in the ocular tissue of other avian species such as chicken, turkey, and quails. This aligns with the human studies showing no significant impact of selenium on cataract prevention [43], signifying that the eyes in poultry, like those in humans, tightly regulate selenium to avoid unnecessary deposits, while Se may help to enhance the antioxidant defenses [44]. Figure 5F presents the selenium content in the breast muscle, which exhibited a non-uniform distribution among treatment groups, indicating moderate variation in selenium deposition. The red selenium at a low dose showed the highest deposition, significantly differing from the lowest selenium level observed in the high-dose red selenium group. The control and both grey selenium groups (T3, T4) exhibited intermediate values without significant differences among them. These results suggest that a lower dose of red selenium may be more efficiently incorporated into muscle tissue, while higher doses could trigger regulatory mechanisms that limit excessive selenium accumulation. This may be due to the muscle’s capacity to store selenium reaching a saturation point, beyond which excess selenium is either excreted or redistributed [39,45]. Additionally, previous studies have demonstrated that muscle selenium content is relatively less sensitive to dietary supplementation, as observed in carp [46]. However, nano-selenium is more bioavailable than selenium methionine in certain fish species, such as crucian carp and broilers under varying environmental conditions [47,48,49,50,51,52]. These findings are consistent with the research indicating that selenium accumulation in muscle is regulated to prevent toxicity while ensuring adequate antioxidant defense [53]. The similarity between the control and grey selenium groups may indicate a more balanced uptake and distribution of selenium in the muscle, maintaining tolerance levels.

## 5. Conclusions

This study demonstrates the superior selenium accumulation and growth-promoting effects of red and grey SeNPs in quails, particularly with grey SeNPs in hepatic tissues. Meanwhile, its distribution in other organs was uniformly delivered or under regulated accumulation such as in the eyes and breast muscle. These findings suggest potential applications in poultry feed fortification strategies, pending optimization of dosing and long-term safety assessment. This research supports the future development of targeted nano-supplementation technologies in avian agriculture.

## Figures and Tables

**Table 1:** Ingredients and nutrient composition of the diet.

Feed Ingredients	Inclusion Rate, %
Soybean meal (46% CP)	34.88
Corn	30.37
Wheat	20.00
Sunflower oil	6.79
Limestone	5.64
MCP	1.29
Salt	0.38
DL-Methionine	0.15
Vitamin and mineral premix ^a^	0.50
Nutrient content, %	
Metabolizable energy MJ/kg	12.13
Crude protein	20.0
Calcium	2.50
Available Phosphorus	0.35
Sodium	0.15
Methionine	0.45
Methionine + cysteine	0.75
Lysine	1.08
Threonine	0.74
Leucine	1.59
Isoleucine	0.86
Arginine	1.33
Tryptophan	0.25

^a^ 1 kg premix provided: 1,000,000 NE vitamin A, 200,000 NE vitamin D3, 4900 mg/kg vitamin E, 200 mg vitamin K3, 150 mg vitamin B1, 500 mg vitamin B2, 1200 mg Ca-d-pantothenate, 400 mg vitamin B6, 2 mg vitamin B12, 11 mg biotin, 2502 mg niacin, 60 mg folic acid, 300,000 mg choline chloride, 13,200 mg Zn, 1920 mg Cu, 9612 mg Fe, 13,200 mg Mn, 180 mg I, 42 mg Se, 12 mg Co.

## Data Availability

The data supporting the findings of this study are contained within the article.

## Abbreviations

The following abbreviations are used in this manuscript:

SeNP	Selenium nanoparticle
Se	Selenium
BCF	Blood cellular fraction
BW	Body weight
AFS	Atomic Fluorescence Spectroscopy
HNO_3_	Nitric Acid
HCl	Hydrochloric Acid
GPx	Glutathione peroxidase
